# A New Cytotoxic Friedelane Acid – Pluricostatic Acid – and Other Compounds from the Leaves of *Marila pluricostata*

**DOI:** 10.3390/molecules13112915

**Published:** 2008-11-25

**Authors:** Dionisio A. Olmedo, José L. López-Pérez, Esther del Olmo, Yelkaira Vásquez, Arturo San Feliciano, Mahabir P. Gupta

**Affiliations:** 1 Centro de Investigaciones Farmacognósticas de la Flora Panameña (CIFLORPAN), Facultad de Farmacia, Universidad de Panamá, Apartado 0824-00172, Panamá, República de Panamá. E-mails: ciflorp4@ancon.up.ac.pa (D-A. O.), yvasquez26@hotmail.com (Y. V.); 2 Departamento de Química Farmacéutica, Facultad de Farmacia, CIETUS, Universidad de Salamanca, Campus Miguel de Unamuno, E-37007, Salamanca, España. E-mails: lopez@usal.es (J-L. L-P.), olmo@usal.es (E. O.), asf@usal.es (A-S. F.)

**Keywords:** *Marila pluricostata*, Clusiaceae, Triterpenoids, Cytotoxicity, 2α,3β-dihydroxy-D:A-friedoolean-28-oic acid.

## Abstract

Bioassay-guided fractionation of the dichloromethane extract of the leaves of *Marila pluricostata* led to the isolation of 2α,3β-dihydroxy-D:A-friedoolean-28-oic acid (pluricostatic acid), a new friedelane triterpenoid, (**1**), ten known triterpenoids and three sterols. Their chemical structures were elucidated through spectroscopic analysis. The less polar fractions, on GC/MS analysis and comparison with a MS library, resulted in the identification of twenty four sesquiterpenoids. The new triterpenoid acid **1** showed cytotoxicity against the MCF-7, H-460, and SF-268 human cancer cell lines with GI_50_ values from 1.2 to 3.3 μg/mL.

## Introduction

**Figure 1:**
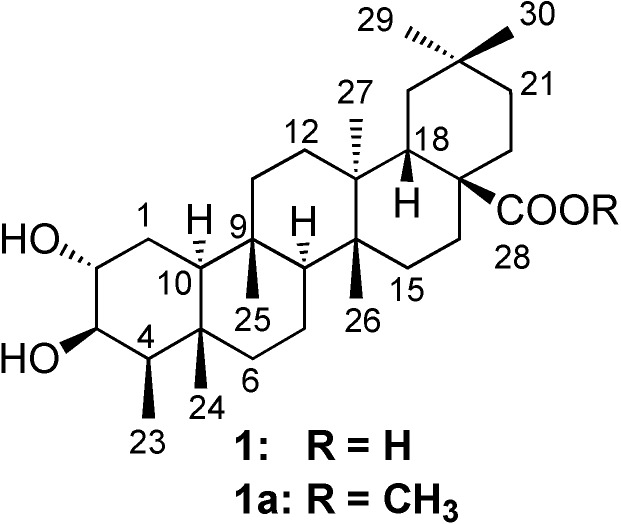
Structures of compound **1** and **1a**.

*Marila pluricostata* Standl. & L.O. Williams (Clusiaceae) is a tree which has a restricted distribution in Panama [1,2,3], Costa Rica [1,2], and Colombia [4]. There is no common name nor any ethnomedical use reported for this species, while *Marila tomentosa* is used in Colombia for the treatment of dysentery [5]. Previous phytochemical studies on *Marila pluricostata* by our group reported several 4-phenylcoumarins [6], some of them showed some degree of antineoplastic cytotoxicity [6], while others displayed transcription inhibitory activity of the HIV Tat receptor [7]. We now report the isolation and structure assignment of one new triterpenoid acid – 2α,3β-dihydroxy-D:A-friedoolean-28-oic acid (**1**) – and the identification of 10 known triterpenoids **2-11**, three sterols **12-14** and 24 sesquiterpenoids as components of its dichloromethane extract. The cytotoxic activity of compound **1** (Figure 1), against three human cancer cell lines (H-460, MCF-7, and SF-268) has also been assessed.

## Results and Discussion

**Figure 2:**
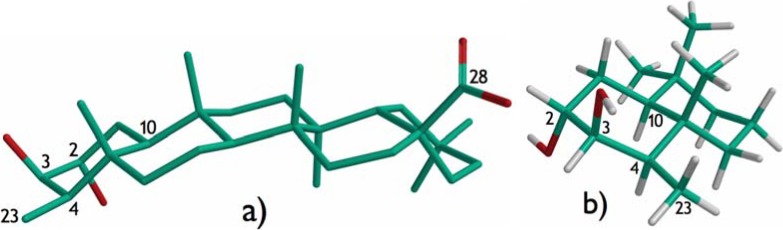
a) Low-energy conformer and full geometry optimization of compound **1**; b) Partial representation of rings A and B for compound **1**.

Compound **1** was isolated as a white amorphous solid. HRFABMS showed [M]^+^ at m/z 474.7162, corresponding to the molecular formula of C_30_H_50_O_4_, typical for a triterpenoid. The IR spectrum exhibited one broad absorption band due to a hydroxyl function (3,410 cm^−1^) and another intense band at 1,688 cm^−1^ corresponding to a carboxylic acid group. The skeletal basis of a friedelane triterpenoid skeleton in compound **1** was deduced from an iterative search carried out within our NAPROC-13 database [8]. This finding was also corroborated by the presence in the ^1^H-NMR spectrum of a doublet (*J* = 7.3 Hz) centered at δ 0.77 ppm, characteristic of the methyl group at position C-23 of friedelane compounds. The search in NAPROC-13 also revealed the presence of a carboxyl group (δ 180.0 ppm) at position C-28 of the friedelane skeleton. Nevertheless, the overlapping of a number of signals in the ^1^H-NMR spectrum did not allow us to perform the complete analysis of many hindered signals, important for the structure assignment. Consequently, some 2D-NMR experiments (HMQC, COSY, HMBC and ROESY) were run in order to obtain more information about the compound. The ^1^H-NMR spectrum showed a double doublet at δ 3.64 (*J*_1_ = 7.8 Hz; *J*_2_ = 2.4 Hz) and a triplet at δ 3.23 (*J* = 2.4 Hz) corresponding to two oxygenated methines, that simultaneously were correlated in the COSY spectrum, cross-linked in the HMQC spectrum with their corresponding carbon signals at δ 70.1 and 74.7 ppm, respectively, and long-range interconnected in the HMBC spectrum. Therefore, both hydroxylated methines must be vicinal. Additionally, in the HMBC spectrum a long-range correlation was observed between the C-23 methyl signal [δ 11.3 ppm] and that of the oxygenated methine proton at 3.64 ppm. These data support the localization of the hydroxyl groups at C-2 and C-3, which were associated with the carbon signals at δ 70.01 [3.64 (*J*_1_ = 7.8 Hz; *J*_2_ = 2.4 Hz)] and 74.7 [3.23 (J = 2.4 Hz)], respectively. Correlations were also observed for the signals of H-1 (1.36 ppm) and those of the methine carbon C-2 and the quaternary carbon at 51.3 ppm, thus assigned to C-10. The signal of the carboxylic carbon (δ 180.0) was correlated with those of the protons of two methylene carbons at 29.4 and 35.4 ppm, thus assigned to C-16 and C-22, respectively. The free carboxylic group signal also correlated with that of the methine carbon at 37.2 ppm, assigned to C-18. These facts confirmed that the carboxyl group was located at position C-28. The remaining connectivities observed in the HMBC spectrum allowed the unambiguous assignment of most ^1^H- and ^13^C-NMR signals. The configuration of the hydroxylated methines C-2 and C-3 were established by ROESY experiments. In the 2D-spectrum a correlation was observed between H-10 and H-4, thus indicating the axial disposition for the latter and, hence, the methyl group at position C-23 must display an equatorial disposition. The coupling pattern of the signal assigned to the proton geminal to the hydroxyl group at position C-3, a triplet with a coupling constant of 2.4 Hz, allowed us to discard the antiperiplanar disposition for H-2 and H-3 because of their observed small coupling constants. The correlations observed in the ROESY spectrum between H-3 and H-4 and H-23 indicated that the proton corresponding to the signal at δ 3.23 ppm was in the alpha disposition, consequently, the hydroxyl group at C-3 must have a beta disposition. Consequently the alpha disposition for the hydroxyl group at C-2 was established. All these statements are in agreement with the structure obtained by molecular modeling studies after a conformational analysis carried out for compound **1** (Figure 2). These data permitted us to establish the absolute configuration of this new compound, which is corroborated by biogenetic pathway for the friedelane type triterpenoids obtained from the squalene oxide, yielding the stereochemistry indicated in this paper. In the literature reviewed there in no mention of any friedelanes of the opposite series.

Accordingly, attempts to obtain the acetonide derivative of **1** by treatment with 2,2-dimethoxypropane in acetone in the presence of acid, such as *p*-toluenesulfonic acid (*p*-TsOH) or trimethylsilyl chloride, left the starting compound **1** unchanged, in agreement with the *trans-*diaxial disposition of the hydroxyl groups. Thus, the compound was identified as the new friedelane 2α,3β-dihydroxy-D:A-friedoolean-28-oic acid (**1**), which we named *pluricostatic acid*. This compound is being reported here for the first time, however, its epimer 2α,3α-dihydroxy-D:A-friedoolean-28-oic acid has been isolated previously from *Euonymus revolutus* [9].

The ten known triterpenoids were identified as: squalene (**2**), friedelin (**3**) [10,11], 4-*epi*-friedelin (**4**) [12], canophyllal (**5**) [13], friedelinol (**6**) [14], canophyllol (**7**) [15,16], 3-oxo-friedelan-28 oic acid (**8**) [9], D:A-friedo-3,4-*seco*-olean-3-oic acid (**9**) [17], *epi*-betulinic acid (**10**) [18], betulinic acid (**11**) [19] and three sterols as β-sitosterol (**12**) [20] stigmasterol (**13**) [21] and 3-*O*-β-glucopyranosyl-sitosterol (**14**) [20] through the complete analysis of their NMR and MS data and comparison with those included in our database NAPROC-13 [8]. NMR data for pluricostatic acid methyl ester (**1a**) are also reported here.

### Biological activity

The dichloromethane extract, fractions and pure compounds were evaluated against three human cancer cell lines, MCF-7 (human breast adenocarcinoma), H-460 (human lung carcinoma) and SF-268 (human CNS carcinona), using the sulphorhodamine B assay [22]. The compounds **1**, **8**-**11** showed GI_50_ values as indicated in the Table 1. The other compounds were considered inactive as their GI_50_ values were higher than 10 μg/mL.

**Table 1:** Antineoplastic cytotoxicity of extracts and compounds **1, 8-11.**

	GI_50_ (μg/mL)		
	MCF-7	H-460	SF-268
dichloromethane extract	5.9	6.2	4.6
methanolic fraction defatted	8.3	7.8	5.5
neutral fraction	1.6	1.5	0.7
acid fraction	2.4	2.7	2.2
**1**	3.0	1.2	3.3
**8**	6.3	5.1	4.6
**9**	7.8	7.2	> 10
**10**	7.2	5.3	6.5
**11**	5.0	3.6	5.1
Adriamycin	0.37	0.42	0.50

## Experimental

### General

Melting points were determined on a Büchi 510-K melting point apparatus and are uncorrected. Optical rotation was determined on an AUTOPOL III automatic polarimeter. IR spectra were obtained on a Nicolet Impact 410 spectrophotometer with KBr pellets. ^1^H-NMR (200 and 400 MHz), and ^13^C- NMR (100 and 50 MHz) spectra were recorded on Brüker AC 200 (200 MHz) and Brüker DRX 400 (400 MHz) spectrometers, with TMS as an internal reference. 2D-NMR spectra were measured with Brüker DRX 400 spectrometer. HRFABMS analysis, a VG-TS250 mass spectrometer (70 eV) was used. Gas chromatography/mass spectrometry (GC/MS) analysis was performed on a Hewlett Packard 5890 serie II, chromatograph apparatus equiped with Hewlett-Packard 5971 mass spectrometer operating in the EIMS-mode at 70 eV and Supelco SPB-1 column (12 m x 0.20 mm, with 0.33 μm film thickness) with helium (10 Psi), as carrier gas at a flow rate of 1 mL/min. The GC oven temperature was kept at 90 ºC for 5 minutes and programmed to 250 ^o^C with a gradient of 2 ^o^C/min and maintained for 10 min at 250 ºC. Injector and detector temperatures were 250 ^o^C and 260 ^o^C, respectively. The injection volume: 1.0 µL of the 10% solution of the fraction A-D. Silica gel (230–400 mesh) for column chromatography and GF_254_ for TLC were obtained from Merck KGaA, 64271 Darmstadt, Germany. Sephadex LH-20 was obtained from Fluka, BioChemika Switzerland. The relative percentages of the identified components were computed from GC peak area on SPB-1 without any correction factor. The minimum source temperature was 90ºC; acquisition mass range, m/z 40-600.

### Plant Material

Leaves of *M. pluricostata* Standl. & L.O. Williams were collected from Llano-Cartí in the province of Panama, Republic of Panama, in November of 2000. Its taxonomic identity was established by Mireya Correa, Director the Herbarium of the University of Panama. Voucher specimens (F-4740 and F-4937) were deposited in the Herbarium of the University of Panama (PMA).

### Extraction, Isolation and Identification

The dried and powdered plant material of *Marila pluricostata* (3.4 kg) was extracted with dichloromethane (10 L) at room temperature for five days. Evaporation of the solvent yielded a crude extract (186.5 g), which was further solubilized in hot *n*-hexane (4 L twice) and cooled (-20 ^o^C) overnight, yielding a soluble fraction (136.1 g), which was defatted successively with MeOH (3L twice) and a saturated solution of urea in MeOH (3L twice). The final soluble part (74.5 g) was partitioned with basic aqueous solution (NaOH 4% x 3 times), yielding an acid fraction (3.0 g) and neutral fraction (71.1 g). The neutral fraction was successively fractionated on a silica gel column using a gradient solvent system *n*-hexane, *n*-hexane/EtOAc (95:5 → 50:50), EtOAc, MeOH to give subfractions (A-K). The subfraction A-D (0.81, 1.03, 0.30 and 1.62 g) were analyzed by GC/MS and 24 known sesquiterpenes were identified by comparison of their retention times and mass spectra (Table 2) with those stored in the spectrometric electronic library (Wiley 275.l) and those reported in the literature [23].

Workup of subfraction E (6.92 g) by repeated CC over silica gel, using *n*-hexane, *n*-hexane/ dichloromethane (95:5 → 60:40), CH_2_Cl_2_, MeOH) led to the isolation of triterpenoids **3** (46.4 mg, 0.024 %), **4** (28.9 mg, 0.015 %), **5** (333.2 mg, 0.780 %) and **6** (23.2 mg, 0.012 %). Subfraction F (29.96 g) was further fractionated on silica gel using *n*-hexane/EtOAc (95:5 → 50:50), EtOAc, MeOH) to yield the triterpenoids **7** (139.1 mg, 0.010 %), and **8** (8 mg, 0.004 %). Subfraction G (5.87 g) on chromatography over silica gel using CH_2_Cl_2_-Et_2_O (80:20) as eluent afforded triterpenoid **9** (118 mg, 0.100 %). Subfraction H (11.32 g) on CC over Sephadex LH 20 using *n*-hexane-CH_2_Cl_2_-MeOH (2:1:1), as eluent gave triterpenoids **10** (115 mg, 0.061 %) and **11** (118 mg, 0.100 %). Subfraction I (11.32 g) by repeated CC on Sephadex LH 20, using *n*-hexane-CH_2_Cl_2_-MeOH (2:1:1) furnished sterols **12** (120 mg, 0.064 %) and **13** (154 mg, 0.082 %). Subfraction J (7.14 g) on CC over silica gel using CH_2_Cl_2_-MeOH (1:1), afforded the new friedelane acid **1** (78 mg, 0.042 %). Finally, subfraction K (6.11 g) on silica gel CC using CH_2_Cl_2_-MeOH (1:1) afforded sterol **14** (39 mg, 0.021 %). 

**Table 2:** Chemical composition of fractions A-D obtained by GC/MS *^a^*.

Components	Retention time (*Rt*) (min.)	Relative (%)	M^+^ *m/z*
fraction A			
α-copaene	17.33	2.6	220
β-caryophyllene	20.01	29.9	204
zingiberene	20.83	1.1	204
germacrene-d	21.84	0.6	204
α-amorphene	23.02	11.7	204
β-selinene	23.19	3.8	204
calarene	23.71	4.1	204
α-muurolene	24.17	3.6	204
χ-cadinene	24.87	7.7	204
calamenene	25.01	1.6	204
δ-cadinene	25.42	3.6	204
α-cadinene	26.09	3.0	204
calacorene	26.85	1.3	200
α-caryophyllene oxide	27.90	10.9	220
Total compounds identified		85.5	
fraction B			
α-copaene	17.37	36.0	220
β-elemene	18.41	0.9	204
β-caryophyllene	20.03	13.4	204
β-cubenene	20.25	3.0	204
α-humulene	21.18	0.8	204
α-amorphene	23.05	11.7	204
α-aromadendrene	23.11	3.8	204
Total compounds identified		69.6	
fraction C			
α-cubebene	16.06	5.1	204
α-copaene	17.35	38.2	220
β-bourbonene	17.76	7.6	204
β-cubenene	20.25	11.3	204
α-aromadendrene	23.13	0.7	204
β-sesquiphellandrene	25.34	0.9	204
Total compounds identified		36.4	
fraction D			
β-farnesene	16.48	2.8	204
α-curcumene	16.92	1.8	202
β-bisabolene	17.72	1.9	204
Squalene	42.40	89.9	410
Total compounds identified		96.4	

*^a^*Quality 95 to 99 %.

*2α,3β-Dihydroxy-D:A-friedoolean-28-oic acid* (**1**): White amorphous solid (CHCl_3_/MeOH): mp 310-313 ºC; [α]_D_^25^ +2.0^o^ (*c*.1.0, MeOH); IR (CHCl_3_) υ_max_ 3410, 2933, 2871, 1688, 1431, 1388, 1247 cm^–1^; ^1^H-NMR (DMSO-d_6_, 400 MHz) δ 3.64 (1H, dd, *J*_1_ = 7.8 Hz; *J*_2_ = 2.4 Hz, H-2), 3.23 (1H, t, *J* = 2.4 Hz, H-3), 2.36 (1H, dd, *J*_1_ = 9.8 Hz, *J*_2_ = 2.4 Hz, H-18), 2.22 (1H, dd, *J*_1_ = 7.8 Hz, *J*_2_ = 4.2 Hz, H-16α), 1.59 (1H, m, H-6β), 1.50 (1H, m, H-16β), 1.40 (1H, m, H-4), 1.37 (1H, m, H-10), 1.36 (2H, m, H-22; 1H, m, H-1β), 1.35 (1H, t, H-21β), 1.34 (1H, t, H-12β), 1.29 (1H, d, H-19β), 1.28 (1H, d, H-1α), 1.26 (2H, m, H-7; 1H, m, H-12α), 1.15 (1H, m, H-21α), 1.12 (1H, m, H-8), 1.10 (1H, m, H-19α), 1.08 (2H, m, H-15; 2H, m, H-11), 0.99 (3H, s, H-30), 0.94 (3H, s, H-27), 0.89 (3H, s, H-29), 0.88 (1H, m, H-6α), 0.82 (3H, bs, H-24), 0.77 (3H, d, J = 7.3 Hz, H-23), 0.76 (3H, s, H-25), 0.74 (3H, m, H-26); ^13^C-NMR (DMSO-d_6_, 100 MHz) δ 180.0 (CO, COOH, C-28), 74.7 (CHOH, C-3), 70.1 (CHOH, C-2), 52.8 (CH, C-8), 51.3 (CH, C-10), 43.9 (C, C-17), 43.0 (CH, C-4), 41.3 (CH_2_, C-6), 38.4 (C, C-13), 37.4 (C, C-14), 37.3 (C, C-5), 37.2 (C, C-18), 36.3 (C, C-9), 35.5 (CH_3_, C-30), 35.4 (CH_2_, C-22), 34.7 (CH_2_, C-11), 34.6 (CH_2_, C-19), 32.5 (CH_2_, C-21), 32.4 (CH_2_, C-15), 30.8 (CH_2_, C-12), 29.8 (CH_3_, C-29), 29.4 (CH_2_, C-16), 28.2 (C, C-20), 23.3 (CH_2_, C-1), 20.0 (CH_3_, C-26), 18.3 (CH_3_, C-27), 17.7 (CH_3_, C-25), 17.1 (CH_2_, C-7), 15.9 (CH_3_, C-24), 11.3 (CH_3_, C-23); HRFABMS m/z 474.7162 (calcd. for C_30_H_50_O_4_, 474.7201).

*Methyl 2α,3β-dihydroxy-D:A-friedoolean-28-oate* (**1a**): Cooled ethereal diazomethane solution (5 mL) was added dropwise to a cooled solution of D:A 2α,3β-dihydroxy-friedoolean-28oic acid (30 mg) in 4 mL of diethyl ether and the reaction mixture was maintained at 0 ºC for 5 h, then concentrated *in vacuo* to give **1a** (32 mg). White amorphous solid (CHCl_3_/MeOH); [α]_D_^25^ +0.0^o ^ (*c*.1.0, MeOH); ^1^H- NMR (CDCl_3_/CD_3_OD 9:1, 200 MHz) δ 3.84 (1H, dd, *J*_1_ = 7.8 Hz, *J*_2_ = 2.4 Hz, H-2), 3.66 (3H, s, COOCH_3_), 3.44 (1H, t, *J* = 2.4 Hz, H-3), 2.42 (1H, dd, *J*_1_ = 9.8 Hz, *J*_2_ = 2.4 Hz, H-18), 2.32 (1H, dd, *J*_1_ = 7.8 Hz, *J*_2_ = 4.2 Hz, H-16α), 1.71 (1H, m, H-1β), 1.69 (1H, m, H-6β), 1.64 (1H, m, H-16β), 1.50 (H, m, H-4), 1.49 (1H, d, *J*_1_ = 2.4 Hz, H-1α), 1.46 (1H, m, H-22β), 1.44 (1H, t, H-12β; ), 1.43 (1H, m, H-10), 1.42 (2H, m, H-15), 1.41 (1H, m, H-22α), 1.38 (3H, s, H-25), 1.36 (1H, m, H-8; 1H, m, H-19β), 1.31 (1H, m, H-12α),1.22 (1H, m, H-11β), 1.17 (2H, t, *J* = 2.4 Hz, H-21), 1.16 (1H, t, *J* = 2.4 Hz, H-19α), 1.14 (1H, m, H-11α), 1.02 (3H, bs, H-26; 3H, bs, H-30), 0.97 (1H, m, H-6α), 0.91 (3H, s, H-29), 0.90 (3H, s, H-24), 0.88 (3H, d, *J* = 7.3 Hz, H-23), 0.84 (3H, s, H-27; 1H, s, H-7β), 0.81 (1H, s, H-7α), ^13^C-NMR (CDCl_3_/CD_3_OD 9:1, 50 MHz) δ 182.0 (CO, COOCH_3_, C-28), 74.1 (CHOH, C-3), 69.5 (CHOH, C-2), 51.6 (CH_3_, COOCH_3_), 51.7 (CH, C-8), 50.1 (CH, C-10), 43.1 (C, C-17), 42.1 (CH, C-4), 39.9 (CH_2_, C-6), 37.2 (C, C-13), 36.3 (C, C-18), 36.2 (CH, C-14), 36.0 (C, C-5), 35.1 (C, C-9), 34.3 (CH_2_, C-22), 33.4 (CH_2_, C-11), 33.3 (CH_2_, C-19), 32.3 (CH_3_, C-30), 31.0 (CH_2_, C-15), 30.9 (CH_2_, C-21), 29.6 (CH_2_, C-12), 28.1 (CH_2_, C-16), 27.7 (CH_3_, C-29), 26.6 (C, C-20), 21.8 (CH_2_, C-1), 18.4 (CH_3_, C-27), 16.4 (CH_3_, C-26), 15.8 (CH_2_, C-7), 15.7 (CH_3_, C-25), 13.8 (CH_3_, C-24), 8.9 (CH_3_, C-23).

Since ^13^C-NMR data of canophyllal (**5**) have not been reported before, these are included here: ^13^C- NMR (CDCl_3_, 50 MHz) δ 213.0 (CO, C-3), 209.0 (CHO, C-28), 59.2 (CH, C-10), 58.2 (CH, C-4), 52.8 (CH, C-8), 47.7 (C, C-17), 42.0 (C, C-5), 41.5 (CH_2_, C-2), 41.0 (CH_2_, C-6), 38.7 (C, C-13), 37.7 (C, C-14), 37.1 (C, C-9), 36.4 (CH, C-18), 35.4 (CH_2_, C-11), 34.9 (CH_2_, C-19), 34.5 (CH_3_, C-29), 33.4 (CH_2_, C-21), 32.4 (CH_2_, C-15, C-16), 30.6 (CH_2_, C-12), 29.4 (CH_3_, C-30), 28.3 (C, C-20), 28.0 (CH_2_, C-22), 22.3 (CH_2_, C-1), 20.0 (C CH_3_, C-26), 18.8 (CH_3_, C-27), 18.0 (CH_2_, C-7), 17.2 (CH_3_, C-25), 14.3 (CH_3_, C-24), 6.8 (CH_3_, C-23).

### Test for in vitro Antineoplastic Cytotoxicity

The antineoplastic cytotoxicity bioassay was performed against breast (MCF-7), lung (H-460) and CNS (SF-268) human cancer cell lines using the sulforhodamine B assay [22].

### Molecular Modeling

Calculations were performed on a Silicon Graphics Indigo computer. Compounds were built using Macromodel v.4. [24]. Conformational analysis was performed by a Monte Carlo random search. All freely rotating bonds were searched with MM2 [24], minimization to a gradient of less than 0.001 kcal/mol.
